# Comparative Hypothalamic Transcriptome Analysis Reveals Crucial mRNAs, lncRNAs, and circRNAs Affecting Litter Size in Goats

**DOI:** 10.3390/genes14020444

**Published:** 2023-02-09

**Authors:** Shucan Dong, Biwei Hou, Chuang Yang, Yaokun Li, Baoli Sun, Yongqing Guo, Ming Deng, Dewu Liu, Guangbin Liu

**Affiliations:** Guangdong Laboratory for Lingnan Modern Agriculture, College of Animal Science, South China Agricultural University, Guangzhou 510642, China

**Keywords:** litter size, goat, lncRNAs, circRNAs, fertility

## Abstract

Litter size is an important indicator to measure the reproductive performance of goats, which is affected by the reproductive function of animals. The hypothalamus, as the regulatory center of the endocrine system, plays an important role in the reproduction of female animals. Here, we performed high-throughput RNA sequencing using hypothalamic tissue from high-fecundity and low-fecundity Leizhou goats to explore critical functional genes associated with litter size. Differentially expressed mRNA, lncRNA, and circRNAs were screened using DESeq and were enriched, and then analyzed by Gene Ontology and Kyoto Encyclopedia of Gene and Genome. Results showed that some of these differentially expressed mRNAs could be enriched in reproductive processes, jak-STAT, prolactin signaling pathway, and other signaling pathways related to reproduction, such as *SOCS3*. Furthermore, the central proteins POSTN, MFAP5, and DCN from protein–protein interaction may regulate animal reproductive activity by affecting cell proliferation and apoptosis. lncRNA MSTRG.33887.2 as well as circRNAs chicirc_098002, chicirc_072583, and chicirc_053531 may be able to influence animal reproduction by participating in folate metabolism and energy metabolism homeostasis through their respective target genes. Our results expand the molecular mechanism of hypothalamic regulation on animal reproduction.

## 1. Introduction

The litter size of goats is an important economic index to measure the production performance of goats, which affects the economic benefit of farmers [1]. The litter size of livestock can be increased by improving the level of feeding and management, but the influence of these strategies is limited [2]. Improving the fecundity of livestock is the key to select excellent varieties with a large number of offspring by genetic breeding. With the development of high-throughput sequencing, variety selection has entered the era of molecular breeding, and many candidate genes related to animal litter size have been excavated continuously [3,4,5]. Leizhou goats primarily live in Leizhou Peninsula in Guangdong Province, China, and they have excellent characteristics such as early sexual maturity and high reproductive capacity [6]. Therefore, the study of the molecular mechanism of high-fertility Leizhou goats is useful to obtain comprehensive understanding of the reproductive process of animals and to improve the efficiency of goat industry.

The litter size of livestock is closely related to the number of mature oocytes excreted by the dam in each reproductive cycle [7]. Ovulation in livestock is strictly regulated by the hypothalamus–pituitary–ovary (HPO) axis [8]. As the initial organ of the HPO axis, the hypothalamus can regulate the secretion of luteinizing hormone (LH) and follicle-stimulating hormone from the pituitary by secreting gonadotropin-releasing hormone (GnRH), thereby controlling gonadal development and sex hormone secretion [9]. In addition, the hypothalamus can influence animal reproduction by coordinating energy metabolism and biological rhythms [10,11,12]. Therefore, the hypothalamus is essential for animal reproduction. However, although the hypothalamus is essential for animal reproduction, little is known about the molecular mechanism by which the hypothalamus regulates animal reproduction.

The regulation of animal reproduction by the hypothalamus requires the participation of coding RNA and non-coding RNA. Previously, non-coding RNAs (ncRNAs) were considered as transcriptional noise. However, at present, many studies have shown that ncRNAs have a remarkable variety of biological functions, regulating gene expression at the level of transcription, RNA processing, and translation [13,14]. A previous study has revealed the mRNA–microRNAs network in the hypothalamus that affects litter size in goats by hypothalamic transcriptomics, but information on long non-coding RNAs (lncRNAs) and circular RNAs (circRNAs) is scarce [15]. circRNAs are ncRNAs that are widely expressed in organisms, which play a role in the regulation of gene expression through circularization and splicing against one another, and their expression is specific to tissue and developmental stages [16,17]. circRNAs are associated with the occurrence of many diseases, but their regulatory mechanisms for reproduction in the hypothalamus are lacking [18]. lncRNAs, a class of non-encoded proteins larger than 200 bp, are considered as key regulators of various biological functions [19]. These coding RNAs interact with ncRNAs to form a complex regulatory network. Investigating this network has important implications for understanding the regulation of reproduction by the hypothalamus. RNA sequencing (RNA-Seq) can obtain almost all mRNA and ncRNA sequence information and expression information of a specific tissue or cell; thus, it is widely used in the mining of molecular biological functions [20,21]. The use of RNA-Seq to mine differential mRNA, lncRNA, and circRNA in the hypothalamus of high- and low-fertility goats is of great significance for a comprehensive understanding of the gene networks in the hypothalamus, which regulate animal reproduction.

At present, information on the regulation of reproduction by circRNA and lncRNA in the hypothalamus of goats is lacking. Thus, we used RNA-Seq to analyze the differential expression of mRNAs (DE mRNAs), lncRNAs (DE lncRNAs), and circRNAs (DE circRNAs) in the hypothalamus of Leizhou goats with high and low fecundity and predicted the network interaction of DE lncRNA and DE circRNA with mRNA. Our research contributes to a comprehensive understanding of the regulatory role of the hypothalamus in animal reproduction.

## 2. Materials and Methods

### 2.1. Animals and Sample Collection

Seven healthy Leizhou goat of similar age and body condition were selected and divided into the high-fecundity group (n = 3) and low-fecundity group (n = 4) based on the litter size. The high-fecundity group had more than one lamb per litter, whereas the low-fecundity group had only one lamb, and three lambs were born to these goats. The female goats were injected with 0.1 mg of cloprostenol to induce estrus [22,23]. After 2 days of injection, the male goat test method (the vas deferens has been ligated) and vaginal observation method were used to check whether the female goat was in heat, and the second estrus identification was carried out on the test female goat at about 18 days. All female goats received estrus synchronization. After the second confirmation of estrus, these seven goats were slaughtered and dissected simultaneously within 24 h to collect hypothalamic tissue. The collected hypothalamus was immediately washed with PBS, after which it was placed in liquid nitrogen for short-term storage and transport, and finally stored at −80 °C [15].

### 2.2. Extraction of Total RNA, Construction of cDNA Library, and Transcriptome Sequencing

Trizol (Thermo Fisher Scientific, Waltham, MA, USA) was used to extract total RNA from the hypothalamus, following the manufacturer’s instructions. NanoDrop ND-2000 (Thermo Science, Wilmington, DE, United States) was used to measure RNA concentration and purity. OD A260/A280 (> 1.8) and A260/A230 (>1.6) were used to assess the purity of RNA, and agarose gel electrophoresis was used to detect the integrity of RNA. After passing the abovementioned indexes, 3 μL of total RNA was selected from each sample for the construction of cDNA library. Paired-end sequencing of these libraries was performed using Next-Generation Sequencing based on the Illumina HiSeq sequencing platform. cDNA library construction and transcriptome sequencing were performed by Shanghai Personalbio Technology Co., Ltd. (Shanghai, China).

### 2.3. Quality Control, Alignment, and Quantification of RNA-Seq Data

The sequencing data contained some adapters and low-quality reads, and these sequences could have caused great interference with subsequent information analysis; thus, further filtering of the sequencing data was required. Our filtering condition was as follows: the joints at the 3′ end were removed by Cutadapt, and the read with an average mass fraction lower than Q20 was also removed. Reference genome indexes were built by Bowtie2, and then filtered Reads were compared with the goat reference genome (Capra_hircus.ARS1.DNA.toplevel.fa) using Tophat2. The data source for the reference genome is the Ensembl database (http://www.ensembl.org/, accessed on 28 August 2018). The transcript of each sample was assembled from the mapped reads by StringTie based on the annotated transcript file from ENSEMBL.

We identified all the clipped forms of transcripts in accordance with StringTie and used gffcompare to compare with the reference genome to find new transcript regions. We identified all sheared forms of transcripts based on StringTie and used gffcompare to compare with the reference genome and identify new transcript regions. The coding potential of new transcripts was predicted using Coding-Non-Coding-Index, Pfam Scan, and CPC to screen for new mRNAs or lncRNAs. Subsequently, fragments per kilobase transcript per million mapped fragments of mRNAs and lncRNAs were calculated by StringTie. When identifying circRNA, the 20 bp at both ends of the unaligned reads in the Tophat2 alignment results was used as anchor sequences to be realigned onto the genome using Bowtie2 for the detection of circRNAs. After aligning the anchor sequence of each sample to the reference genome, we combined the alignment results of all samples to identify circRNAs using find_circ. Then, the highly confident circRNAs were filtered on the basis of the following criteria: (1) breakpoints = 1; (2) anchor_overlap ≤ 2; (3) edit ≤ 2; (4) n_uniq > 2 and n_uniq > samples and n_uniq > int (1/2 samples); (5) best_qual_A > 35 or best_qual_B > 35; (6) circRNA is less than 100 k in length. The expression level of circRNAs was estimated by transcript per million.

### 2.4. Analysis of Differentially Expressed Transcripts

The mRNAs, lncRNAs, and circRNAs expressed differentially between the high-fecundity group and low-fecundity group were identified by DESeq, and the conditions for screening differentially expressed genes were as follows: expression difference multiplicity |log_2_ fold change| > 1, significance *p*-value < 0.05.

### 2.5. Analysis of lncRNA-Regulated Target Genes

The function of lncRNAs is primarily achieved by acting on target genes through cis- and trans-actions. The cis-acting target gene prediction suggested that the function of lncRNA was related to protein-coding genes close to its coordinates. The basic principle of trans-action target gene prediction was that the function of lncRNA was not related to the position relationship of the coding gene but to the protein-coding gene it co-expresses. Therefore, we searched for genes encoded by the nearest protein of the lncRNA gene and concluded that the protein-coding gene we found may be its corresponding cis-regulatory target gene [24]. Given the small number of samples, lncRNA trans-regulatory function prediction cannot be made.

### 2.6. Protein–Protein Interaction (PPI) Network Construction

The STRING database (https://cn.string-db.org, accessed on 9 November 2022) was used to predict the potential interactions among proteins translated from mRNAs, and a confidence score ≥ 0.4 was selected. The PPI network was visualized through Cytoscape (v3.8.0, http://www.cytoscape.org/, accessed on 15 November 2022). 

### 2.7. GO and KEGG Pathway Analysis

Functional analysis of differential genes was performed using the GO and KEGG enrichment analysis tools of Omicshare Tools (https://www.omicshare.com/tools, accessed on 9 October 2022) from Gene Denovo Biotechnology Co. Ltd. (Guangzhou, China) *p* < 0.05 was considered significant.

### 2.8. Quantitative Real-Time PCR (qRT-PCR) Validation

We randomly selected two DE mRNAs, two DE lncRNAs, and two DE circRNAs for qRT-PCR to verify the reliability of the RNA-seq data. The RT Reagent Kit with gDNA Eraser (Takara, Dalian, China) was used for total RNA reverse transcription to synthesize cDNA, according to the manufacturer’s instructions. PowerUp SYBR Green Master Mix (Life ABI, Austin, TX, USA) and QuantStudio 5 (Life ABI, Austin, TX, USA) were used to perform qRT-PCR. Melting curve analyses confirmed that all primers were specific for their respective transcript. *Capra hircus β-actin* were used as internal controls, and the results were calculated using the 2^−ΔΔCt^ method [25]. All the primers were synthesized by Sangon Biotech Co., Ltd. (Shanghai, China). The values were expressed as means ± SD. The primer sequences are shown in Table S1.

## 3. Results

### 3.1. Results of Sequencing and Characteristics of Transcripts

In total, seven hypothalamic tissue samples obtained from high- and low-fecundity Leizhou goats were subjected to Illumina sequencing after rRNA depletion, which led to the generation of approximately 103.30 million raw reads per sample. After quality control, approximately 102.30 million clean reads per sample were obtained, accounting for 99.03% ± 0.28% of raw reads in each library. The Q30 ratio was >91%, and no GC bias was observed, indicating that sequencing clean data were qualified. In the alignment of all clean reads, 87.28% ± 1.02% of the clean reads was perfectly mapped to the reference genome of *chx*, including 97.84% ± 0.33% uniquely mapped, indicating that sequencing data had an excellent performance and high credibility for downstream analysis results (Table S2). After assembly, a total of 20,194 mRNAs, 3895 lncRNAs, and 37,655 circRNAs were identified in the hypothalamus of Leizhou goats among seven samples (Table S3).

### 3.2. Differential Transcription Expression Profile

**Figure 1:**
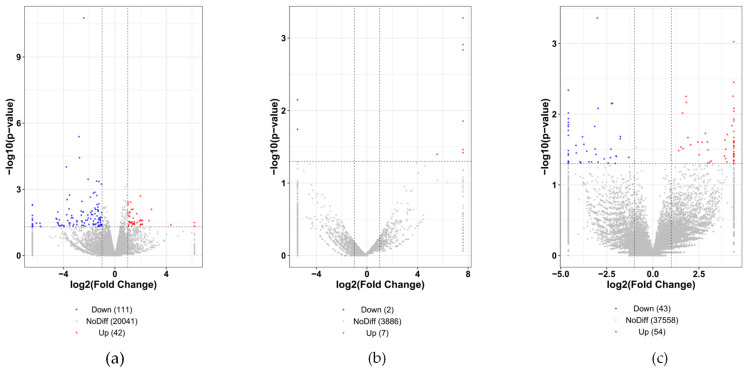
Analysis of differentially expressed mRNAs, lncRNAs, and circRNAs. Volcano plot of DE mRNAs (**a**), DE lncRNAs (**b**), and DE circRNAs (**c**). Red and blue indicate the upregulated and downregulated expression levels, respectively.

In the hypothalamus, as an important organ of the HPO axis in female mammals, the differential expression of its genes or transcripts may affect the fecundity of Leizhou goats. Therefore, we screened DE mRNAs, DE lncRNAs, and DE circRNAs from RNA-seq data using *p* ≤ 0.05 and |log_2_ fold change| ≥ 1 as screening conditions. A total of 153 DE mRNAs, 9 DE lncRNAs, and 97 circRNAs were identified and compared between the high- and low-fecundity group. The volcano plot showed that a total of 42 mRNAs, 7 lncRNAs, and 54 circRNAs were significantly upregulated, whereas 111 mRNAs, 2 lncRNAs, and 43 circRNAs were significantly downregulated in the high-fertility group of Leizhou goats compared with the low-fertility group of Leizhou goats (Table S4, Figure 1). These differentially expressed transcripts are valuable for further studies to examine the reproductive performance of Leizhou goats.

### 3.3. GO and KEGG Enrichment Analysis of DE mRNAs

**Figure 2:**
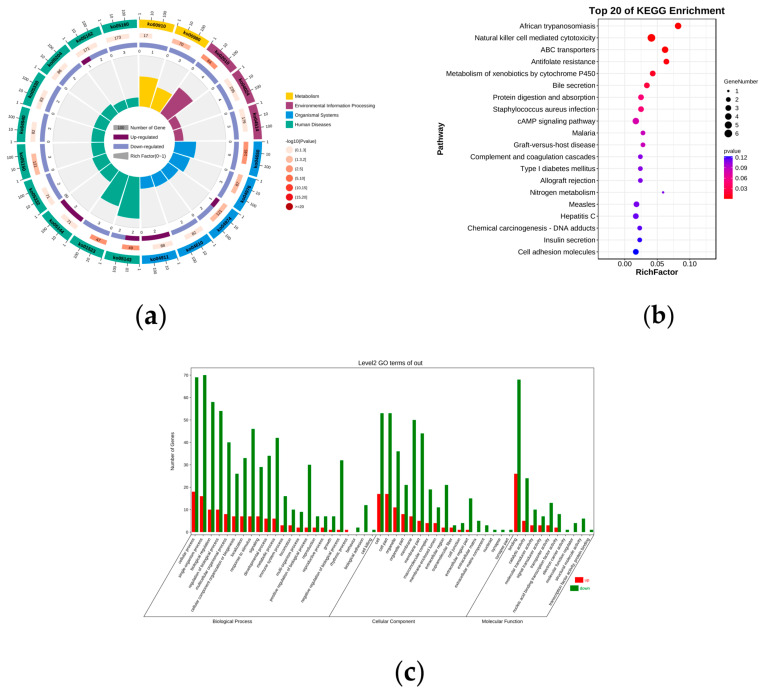
KEGG and GO pathway annotations of DE mRNAs. The enrichment circle map (**a**) and enrichment bubble map (**b**) of the top 20 KEGG terms of DEmRNA. The first lap indicates the top 20 KEGG terms, and the number of genes corresponds to the outer lap. The second lap indicates the number of genes in the genome background and Q values for the enrichment of the upregulated genes for the specified BP. The third lap indicates the ratio of the upregulated genes (deep purple) and downregulated genes (light purple). The fourth lap indicates the enrichment factor of each KEGG term. Enrichment factor represents the ratio between the differentially expressed genes and all annotated genes enriched in the pathway. Bubble scale represents the number of different genes; the depth of bubble color represents *p* value. (**c**) GO annotation of up− and down−regulated genes in BP, CC, and MFs. The abscissa and ordinate represent the GO terms and number of enriched genes, respectively.

We performed GO and KEGG enrichment analyses on 153 DE mRNAs to predict the potential biological function of these DE mRNAs. KEGG enrichment analysis showed that these DE mRNAs were significantly enriched to eight pathways, and the three most significant pathways included African trypanosomiasis, natural killer cell-mediated cytotoxicity, and ABC transporters. These signal transduction systems may play a role in the reproductive process, which requires further investigation (Table S5, Figure 2a,b). In GO terms, in the order of the number of enriched genes, the most evident correlation: with biological processes (BP) were cellular process, single-organism process, biological regulation, and regulation of BP; with molecular function (MF) were binding and catalytic activity; and with cellular components (CC) were cell and cell part (Table S6, Figure 2c). Notably, nine genes were enriched into the reproductive process, namely, dpy-19-like 2 (*DPY19L2*), cilia and flagella-associated protein 157 (*CFAP157*), cytokine signaling 3 (*SOCS3*), transcription factor 21 (*TCF21*), keratin 8 (*KRT8*), retinoic acid 6 (*STRA6*), natriuretic peptide precursor-A (*NPPA*), LIM homeobox 3 (*LHX3*), and serpin family F member 1 (*SERPINF1*). These genes may be involved in physiological activities related to reproduction, which are worth studying.

### 3.4. Protein–Protein Interaction Network of DE mRNAs

**Figure 3:**
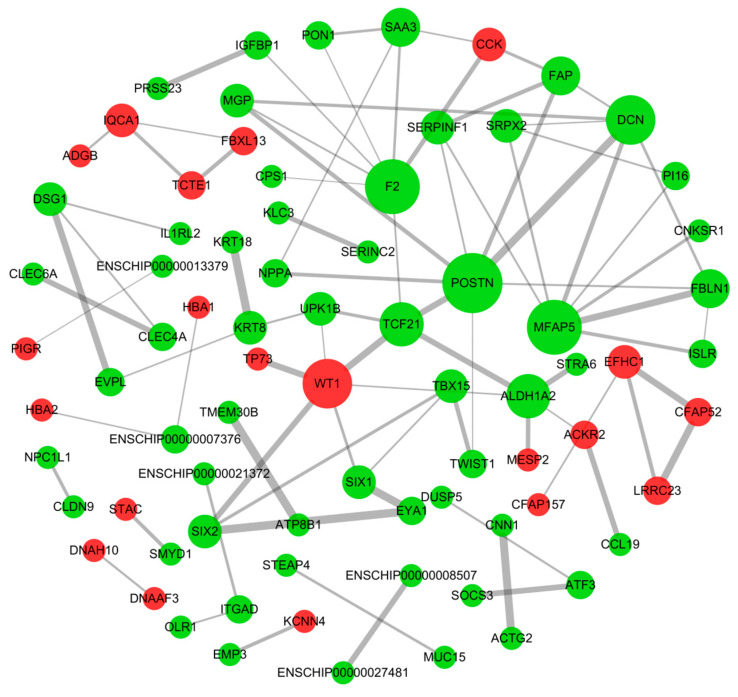
Protein−protein interaction network of DE mRNAs. The nodes in the network represent proteins, and the width of the lines in the nodes indicates the interaction between two proteins. Red nodes, upregulated mRNA; green nodes, downregulated mRNA. The wider the line, the stronger the interaction between the two proteins.

In investigating the important role of protein interactions in high- and low-fecundity Leizhou goats, we performed PPI network analysis on differential mRNA using String (Figure 3). A total of 140 proteins were enriched in these differential mRNA. After removing the proteins that did not interact with one another, 77 proteins were left to form a complex functional network. Based on the PPI diagram, periostin (POSTN, degree = 8), coagulation factor II (F2, degree = 7), microfibril-associated protein 5 (MFAP5, degree = 7), and decorin (DCN, degree = 6) were strongly associated with other proteins (Table S7). These central proteins may play an important function in the regulatory role of the hypothalamus in Leizhou goats.

### 3.5. Functional and Pathway Enrichment Analyses of DE lncRNAs

**Figure 4:**
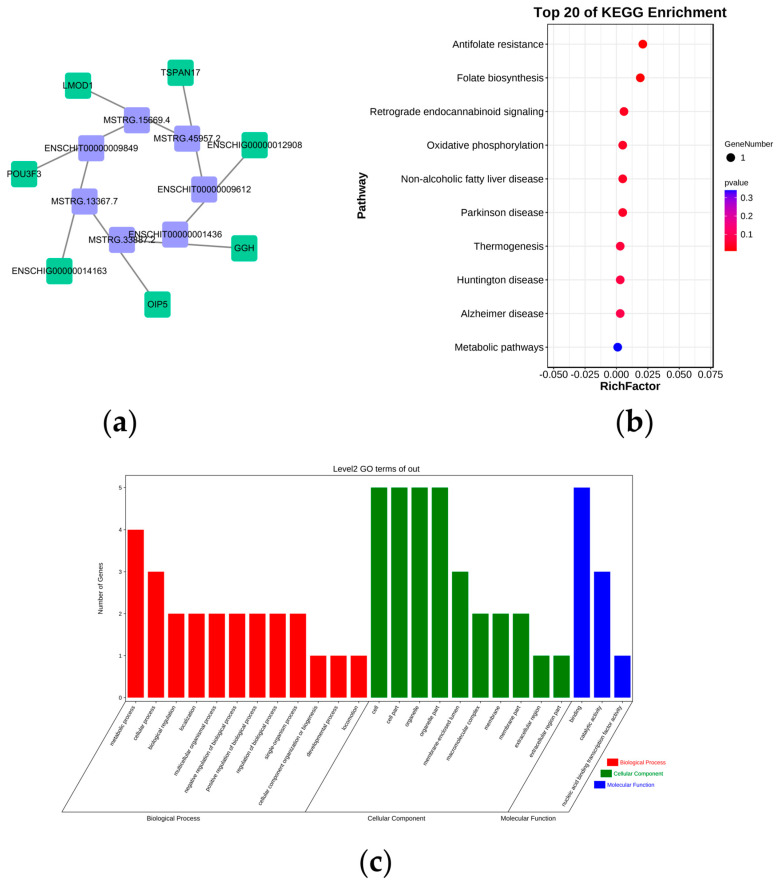
Functional identification of DE lncRNAs. (**a**) DE lncRNA−mRNA network map. Purple represents DE lncRNA; green represents DE lncRNA’s cis-regulatory target genes. (**b**) KEGG pathway analyses of target genes of DE lncRNAs. (**c**) GO analysis of target genes of DE lncRNAs.

lncRNA plays an important role in many physiological activities and diseases. lncRNAs can exert regulatory effects through cis-action and trans-actions. The prediction of cis-acting target genes suggests that the function of lncRNA is related to the protein-coding genes close to its coordinates. Here, we constructed a DE lncRNA−mRNA network for DE lncRNAs and their possible cis-regulatory target genes to explore the function of DE lncRNAs (Table S8, Figure 4a). In exploring the effect of DE lncRNAs in the hypothalamus on the fecundity of Leizhou goats, we analyzed the target genes of DE lncRNAs by KEGG and GO enrichment analyses to predict the potential function of DE lncRNAs. In KEGG enrichment analysis (Table S9, Figure 4b), the DE lncRNAs were enriched to a total of 11 pathways, of which six pathways were significantly correlated: antifolate resistance, folate biosynthesis, retrograde endocannabinoid signaling, oxidative phosphorylation, and non-alcoholic fatty liver disease. GO enrichment analysis showed that most of the BP of the target genes of the DE lncRNAs were enriched into metabolic and cellular processes, and most of the MF of the DE lncRNAs were binding; furthermore, most of the CC of the DE lncRNAs were cell and cell part (Table S10, Figure 4c).

### 3.6. Functional Identification of DE circRNAs

**Figure 5:**
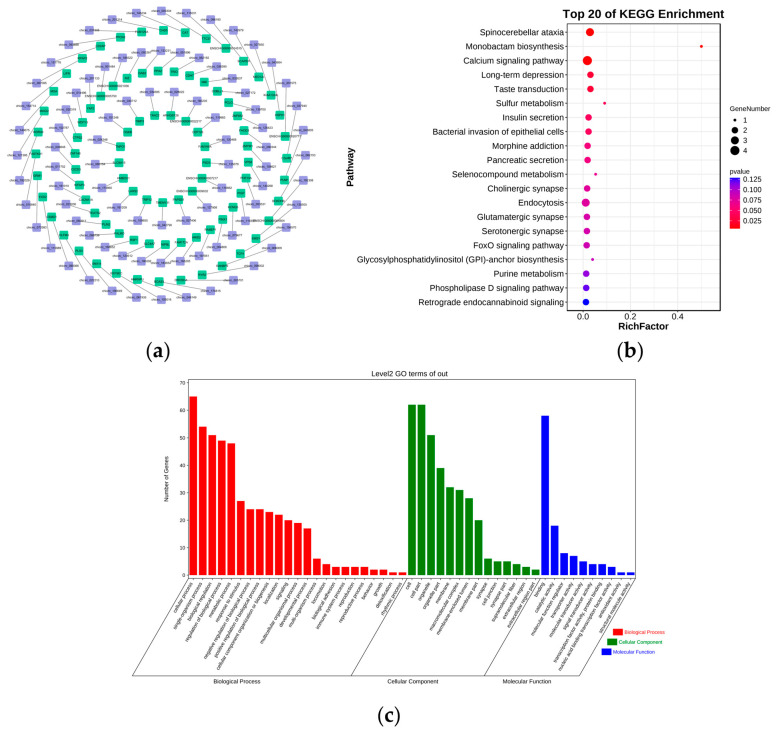
Functional identification of DE circRNAs. (**a**) DE circRNA−mRNA network map. Purple represents DE circRNA, and green represents target genes of DE circRNA. (**b**) KEGG pathway analyses of target genes of DE circRNAs. (**c**) GO analysis of target genes of DE circRNAs.

In exploring the function of DE circRNAs in the hypothalamus of high- and low-fecundity Leizhou goats, we predicted their host genes (Table S11, Figure 5a) and performed KEGG and GO enrichment analyses on the predicted host genes. The KEGG results showed that the top four significantly associated pathways were spinocerebellar ataxia, monobactam biosynthesis, calcium signaling pathway, and long-term depression (Table S12, Figure 5b). A total of 269 significant BP, 81 significant CC, and 90 significant MF were found in GO terms. The four BP terms with the most enriched genes were cellular process, single-organism process, biological regulation, and BP regulation. The four MF terms with the most enriched genes were binding, catalytic activity, MF regulator, and transporter activity. The four CC terms with the most enriched genes were cell, cell part, organelle, and organelle part (Table S13, Figure 5c).

### 3.7. Sequencing Data Validation

**Figure 6:**
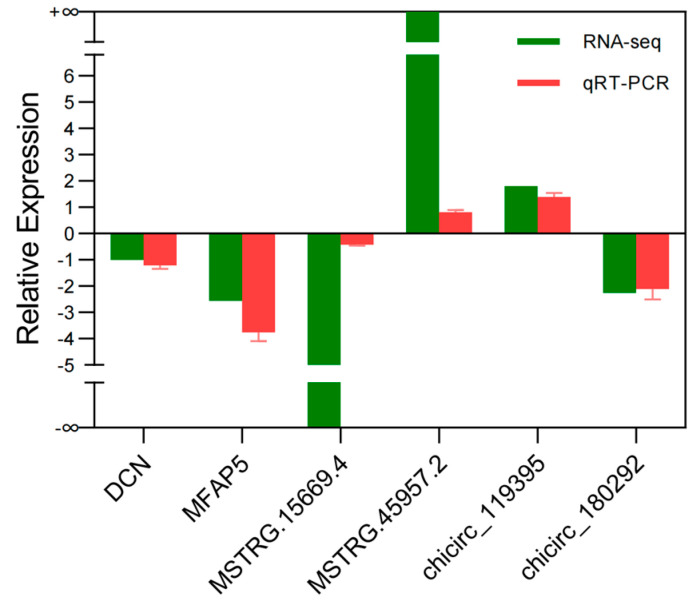
Comparison between RNA−seq and qRT−PCR results. The vertical axis shows the mean of fold change (log_2_ scale) of each RNA measured by qRT−PCR and sequencing.

In verifying the RNA-seq results, we randomly selected several mRNAs, lncRNAs, and circRNAs for expression-level detection. The expression patterns of these genes were determined using qRT-PCR and compared with RNA-seq results. The results showed that the RNA-seq and qRT-PCR results exhibited similar expression patterns (Figure 6), indicating that the data generated by RNA-seq are reliable.

## 4. Discussion

As an important economic livestock, goats provide meat, milk, and fur that greatly enrich human life, and they are particularly important to tropical agricultural systems, sustainable agricultural development, pastoralist poverty reduction, and marginal grazing [26,27]. As an indigenous goat breed in China, the Leizhou goat has excellent reproductive performance. The hypothalamus is the center of the endocrine system and the nervous system because of its ability to receive many nerve impulses that regulate reproduction, growth, and energy metabolism in the animal organism. Therefore, we used RNA-seq to identify DE mRNAs, DE lncRNAs, and DE circRNAs in the hypothalamus of high- and low-fertility Leizhou goats and predicted their functions. Our study will expand the molecular mechanism in the hypothalamus that regulates litter size in goats.

### 4.1. SOCS3 in the Hypothalamus May Affect the Fertility of Leizhou Goats

Animal traits are regulated by gene expression. Litter size is a quantitative character controlled by minor effects and polygenes. With the development of sequencing technology, many genes associated with lambing number were gradually being uncovered, such as bone morphogenetic protein receptor type 1B (*BMPR1B*) [28,29], growth differentiation factor 9 (*GDF9*) [30,31], and bone morphogenetic protein 15 (*BMP15*) [32]. These genes affected the number of lambs by influencing the ovulation rate of follicles and embryo survival. At present, no studies have been conducted on the differentially expressed genes in the hypothalamus of high- and low-fertility Leizhou goats. A total of 20,194 mRNAs were identified in the hypothalamus of high- and low-fertility Leizhou goats, of which the expression of 153 mRNAs was significantly different. These DE mRNAs were significantly enriched into the immune system, membrane trafficking, digestive system, tissue, and organ development. Furthermore, some of the signaling pathways (prolactin signaling pathway and Jak-STAT signaling pathway) enriched by these differential genes were similar to the results of a previous study [15]. Therefore, the prolactin signaling pathway and Jak-STAT signaling pathway in the follicular hypothalamus may influence litter size in goats. Remarkably, we found that SOCS3 was enriched into multiple signaling pathways associated with reproduction, including the prolactin signaling pathway and JAK-STAT signaling pathway. SOCS3 belongs to the SOCS family. The study found that the SOCS family of proteins included negative-feedback inhibitors of signaling induced by cytokines that act via the Jak-STAT pathway [33]. The Jak-STAT signaling pathway was the central node of cell function. SOCS3 could directly and specifically bind to JAK or its receptor to inhibit the kinase activity of JAK, thereby inhibiting the Jak-STAT signaling pathway [34]. The Jak-STAT signaling pathway has important functions in GnRH neurons [35]. Therefore, SOCS3 may be able to influence the function of GnRH neurons by regulating the Jak-STAT signaling pathway. SOCS3 may also be involved in regulating leptin signal transduction to affect the reproductive function of the hypothalamus. Leptin, a key metabolic messenger in the neuroendocrine reproductive axis, coordinates fertility by acting on neurons in the preoptic region of the hypothalamus and inducing the synthesis of the freely diffusible volume-based transmitter NO through the activation of neuronal NO synthase (nNOS) in these neurons [36]. As a negative feedback signal inhibitor, *SOCS3* can affect *JAK* and *STAT* expression to regulate the activity of leptin receptor b, thereby affecting the activity of the leptin signaling pathway [37,38]. Alterations in the activity of the leptin signaling pathways can affect the secretion of proopiomelanocortin (POMC) and Kisspeptin, thereby affecting GnRH secretion, thereby regulating animal reproductive function [39,40]. In addition, a previous study has found that prolactin can affect the reproductive ability of female rats by regulating kisspeptin neurons in the arcuate nucleus [41]. We hypothesize that *SOCS3* may regulate the secretion of reproduction-related hormones by affecting the activity of multiple pathways, such as the JAK-STAT signaling pathway, leptin signaling pathway, and prolactin signaling pathway, which may be a factor contributing to the difference in reproductive capacity of Leizhou goats.

### 4.2. Pivot Protein Function Analysis in High- and Low-Fecundity Leizhou Goats

Among PPI networks, the pivot proteins are encoding products from vital mRNAs, which may play important regulatory roles in the hypothalamus. *POSTN* is a cell-associated protein that participates in cell migration and proliferation, tumorigenesis, and inflammation responses, and it plays an important role in TGF-β signaling [42]. Many genes of the TGF-β family have been found to affect the litter size of sheep, such as *BMP15* [43] and *GDF9* [44]. In addition, *POSTN* regulates spermatogonia proliferation through the Wnt/β-catenin signal pathway, which may serve as a cytokine for male infertility treatment [45]. Therefore, *POSTN* may play a role in regulating the reproductive capacity of Leizhou goats by determining cell fate or influencing certain signaling pathways in the hypothalamus. *MFAP5* is an extracellular matrix glycoprotein, which has been proven to be involved in the signal transduction of microfiber assembly, elastic formation, and cell survival. It plays an important role in the occurrence of breast cancer [46], ovarian cancer [47], and cervical cancer [48]. As a member of the small leucine-rich proteoglycan family of proteins, *DCN* determines cell fate by mediating various cellular processes such as autophagy, inflammation, cell cycle, and apoptosis [49,50,51]. These pivot proteins affect cell survival, but their role in the hypothalamus have not yet been reported. We hypothesize that they may regulate the HPO axis by regulating the activity of some hypothalamic neurons to affect the secretion of reproduction-related hormones. The effect of these central proteins on lambing numbers of Leizhou goats deserves further exploration.

### 4.3. Functional Analysis of DE lncRNAs in High- and Low-Fecundity Leizhou Goats

Gene expression is regulated by ncRNAs such as lncRNAs and circRNAs. Based on the Encyclopedia of DNA Elements transcriptome project, only about 1.2% of the genome can encode proteins, whereas about 80% of the genome is actively transcribed into various ncRNAs [52]. These ncRNAs are involved in a wide range of BPs, such as cell survival, enzyme activity, and disease development. lncRNA and circRNA, as important non-coding RNA family members, have been shown to play a role in the secretion of hypothalamic GnRH. For example, circRNAs such as oar_circ_0018794 and oar_circ_0008291 in sheep may be involved in reproduction by affecting hypothalamic GnRH activity or the expression of key genes [53]. A total of 9 DE lncRNAs and 97 DE circRNAs were identified in high- and low-fecundity Leizhou goats. These nine DE lncRNAs were significantly enriched for antifolate resistance, folate biosynthesis, and retrograde endocannabinoid signaling. Studies have shown that retrograde endocannabinoid signaling is involved in regulating fertility, which reduces GABAergic afferent drive onto GnRH neurons via the activation of presynaptic CB1 receptors [54]. Folate is an important cofactor for DNA synthesis, repair, methylation modification, and other related enzymes necessary for normal cell growth and replication [55]. The appropriate folate concentration promotes G protein subunit α Q (*GANQ*) promoter methylation, thereby affecting GnRH secretion [56]. In addition, different folate intake during pregnancy affects the development and function of two neuron populations in rats after birth, hypothalamic neuropeptide Y, and opioid melantogen (POMC) [57]. Mammals cannot synthesize folate, and they depend on supplementation to maintain normal levels. Therefore, folate absorption and metabolism may affect the function of the hypothalamus. *GGH* can affect cell proliferation, and intracellular folate metabolism is a ubiquitously expressed intracellular enzyme. Based on our sequencing results, *GGH* is a cis-regulatory target gene of lncRNA (MSTRG.33887.2). We hypothesized that lncRNAs (MSTRG.33887.2) may regulate *GGH* expression through cis-regulatory effects to influence hypothalamic folate metabolism. Moreover, these DE lncRNAs were significantly enriched during the development of glomeruli and adrenal glands in GO terms. Glucocorticoids secreted by the adrenal glands can directly regulate the secretion of GnRH in the hypothalamus to influence animal reproduction, although the mechanism of this regulatory effect remains unclear [58]. Furthermore, glucocorticoids secreted by the adrenal glands can indirectly regulate hypothalamic and pituitary reproductive functions by affecting the activity of KISS1 and GnIH neurons [59]. lncRNA has a wide range of biological functions, and it plays an important role in epigenetic modification, transcription, and post-transcriptional regulation. However, given the complexity of its action mechanism, all of its action mechanisms have not been fully understood, which is worthy of further study.

### 4.4. Functional Analysis of DE circRNAs in High- and Low-Fecundity Leizhou Goats

circRNAs are a class of non-coding RNA that can regulate the expression of host genes by competitively splicing pre-RNA with host genes or binding to U1snRNP (U1 small nuclear ribonucleoprotein) through RNA–RNA interaction and then further interacting with the PolII transcriptional complex to enhance host gene expression [60]. Therefore, studying the function of the host genes of circRNAs is important to understand the biological function of circRNAs. Ryanodine receptor 2 (*RYR2*) is the host gene of chicirc_098002, which play a critical role in regulating insulin secretion and glucose metabolism homeostasis [61]. In vitro tests have shown that a high-glucose concentration (450 mg/dL) environment led to irreversible cellular damage in GnRH-secreting neurons, which may cause dysfunction of the hypothalamic GnRH pulse generator [62]. chicirc_098002 was downregulated in highly fertile Leizhou goats, and it may regulate *RYR2* to influence hypothalamic glucose concentration to regulate GnRH secretion. cAMP positively regulated GnRH secretion, and its mechanism of action may stimulate GnRH release by increasing cAMP through depolarization of neurons initiated by increased cation conductance and cAMP-gated cation channels [63,64]. Phosphodiesterase 10A (*PDE10A*) is the host gene for chicirc_053531, which encodes a protein that belongs to the cyclic nucleotide phosphodiesterase family responsible for the hydrolysis of cAMP and cGMP into the inactive forms of 5′-AMP and 5′-GMP, respectively [65]. Therefore, we hypothesize that *PDE10A* may affect GnRH secretion by altering cAMP levels. Our study found that the host gene glutamate metabotropic receptor 1 (*GRM1*) of chicirc_072583 was enriched in multiple reproduction-related pathways, such as glutamatergic synapse, retrograde endocannabinoid signaling, and estrogen signaling pathway. γ-Aminobutyric acid and glutamate play an important role in the control of GnRH neuronal excitability. The co-culture of GnRH neurons and astrocytes can increase *GRM1* expression and GnRH secretion, indicating that *GRM1* may play a role in the secretion of GnRH [66]. In addition, *GRM1* may serve as a potential molecular marker for controlling seasonal reproduction and litter size in sheep, which is similar to our findings. Therefore, these DE circRNAs may play an important role in regulating the reproductive activity of females.

## 5. Conclusions

Our study provides the first network of DE lncRNAs, mRNAs, DE circRNAs, and mRNAs from the follicular phase in high- and low-fecundity Leizhou goats. In addition, we identified several DE mRNAs (*SOCS3*, *POSTN*, *MFAP5*, and *DCN*), DE lncRNAs (lncRNA [MSTRG.33887.2]), and DE circRNAs (chicirc_098002, chicirc_053531, and chicirc_072583) from the RNA-seq data of high- and low-fecundity Leizhou goats. These DE mRNAs, DE lncRNAs, and DE circRNAs may play an important role in the regulation of the hypothalamus during the follicular phase, which was worthy of further investigation into the reproductive regulatory mechanism of lambing in goats in the future.

## Data Availability

The data presented in this study are available upon request from the corresponding author.

## Supplementary Materials

The following supporting information can be downloaded at: https://www.mdpi.com/article/10.3390/genes14020444/s1, Table S1: Primer sequences used for qRT−PCR; Table S2: Summary of the transcriptome sequencing data obtained in this study; Table S3: CircRNAs information detected in sequence data of hypothalamic transcriptome of Leizhou Goat; Table S4: Expression information the different expression gene; Table S5: The KEGG pathway annotations of DE mRNAs; Table S6: The GO pathway annotations of DE mRNAs; Table S7: Protein information in protein-protein interaction network; Table S8: The DE LncRNAs and their possible cis-regulatory target genes; Table S9: The KEGG pathway annotations of target genes of DE lncRNAs; Table S10: The GO pathway annotations of target genes of DE lncRNAs; Table S11: The DE circRNAs and their host genes; Table S12: The KEGG pathway annotations of host genes of DE circRNAs; Table S13: The GO pathway annotations of host genes of DE circRNAs.
